# 

**Published:** 1839-12

**Authors:** 


Vol I.—No. IV.

EDITED BY
CHAPIN A. HARRIS, M. D.
Baltimore.
ELEAZAR PARMLY,
New-York.

PUBLISHING COMMITTEE,
E. PARMLY, | E. BAKER,
S. BROWN.

NEW YORK,

GEORGE ADLARD, 168 BROADWAY,

BALTIMORE,

ARMSTRONG & BERRY.

PRICE---THREE DOLLARS PER ANNUM.
Kelley & Fraetas, Printers,

